# Study protocol of Prednisone in episodic Cluster Headache (PredCH): a randomized, double-blind, placebo-controlled parallel group trial to evaluate the efficacy and safety of oral prednisone as an add-on therapy in the prophylactic treatment of episodic cluster headache with verapamil

**DOI:** 10.1186/1471-2377-13-99

**Published:** 2013-07-28

**Authors:** Dagny Holle, Jan Burmeister, André Scherag, Claudia Ose, Hans-Christoph Diener, Mark Obermann

**Affiliations:** 1 Department of Neurology, University Hospital Essen, Hufelandstraße 55, 45122, Essen, Germany; 2 Center for Clinical Trials, Essen (ZKSE) and Institute for Medical Informatics, Biometry and Epidemiology (IMIBE), University Hospital Essen, Hufelandstraße 55, 45122, Essen, Germany

**Keywords:** Episodic cluster headache, Prednisone, Verapamil, Prophylactic treatment, Clinical trial, Prospective study, Study protocol

## Abstract

**Background:**

Episodic cluster headache (ECH) is a primary headache disorder that severely impairs patient’s quality of life. First-line therapy in the initiation of a prophylactic treatment is verapamil. Due to its delayed onset of efficacy and the necessary slow titration of dosage for tolerability reasons prednisone is frequently added by clinicians to the initial prophylactic treatment of a cluster episode. This treatment strategy is thought to effectively reduce the number and intensity of cluster attacks in the beginning of a cluster episode (before verapamil is effective). This study will assess the efficacy and safety of oral prednisone as an add-on therapy to verapamil and compare it to a monotherapy with verapamil in the initial prophylactic treatment of a cluster episode.

**Methods and design:**

PredCH is a prospective, randomized, double-blind, placebo-controlled trial with parallel study arms. Eligible patients with episodic cluster headache will be randomized to a treatment intervention with prednisone or a placebo arm. The multi-center trial will be conducted in eight German headache clinics that specialize in the treatment of ECH.

**Discussion:**

PredCH is designed to assess whether oral prednisone added to first-line agent verapamil helps reduce the number and intensity of cluster attacks in the beginning of a cluster episode as compared to monotherapy with verapamil.

**Trial registration:**

German Clinical Trials Register DRKS00004716

## Background

Episodic cluster headache (ECH) is a primary headache disorder characterized by intense unilateral attacks of facial and head pain lasting between 15 and 180 minutes accompanied by trigemino-autonomic symptoms. The attacks occur between once every other day and eight times per day for an episode lasting between a week and several months. Headache episodes (“inside bouts”) are followed by symptom-free intervals (“outside bouts”) with duration of at least one month. These episodes follow a circadian and circannual rhythm [1].

Therapy for ECH consists of abortive medication such as high flow oxygen, triptans, analgesics or intranasal lidocaine and prophylactic treatment to reduce the number of attacks and quickly end the bout, e.g. verapamil, topiramate or lithium [2-4].

Amongst these, verapamil is regarded as the drug of choice by national and international therapy guidelines. In Germany verapamil is recommended by the “Gemeinsamer Bundesausschuss” (“Federal Joint Committee”) as an off-label-indication (meaning that the treatment is reimbursed by the sick funds). Verapamil has shown efficacy in some randomized controlled studies [5]. Due to its delayed onset of efficacy of usually 10 to 14 days and the required slow titration of the dose to increase tolerability, guidelines recommend initiating short-term prophylactic treatment with overlapping prednisone administration [2-4].

### Rationale for a phase III trial

Studies investigating the efficacy of prednisone in ECH do not yet provide clear evidence in favor of this therapy [6-12]. While several small studies concluded that there is no prednisone effect in ECH patients [6,13-15], other studies suggested that prednisone has an effect in reducing the number of attacks and even terminating the bout [7,9,11]. In a non-randomized trial, 14 ECH patients were administered 250 mg methylprednisolone I.V. over the course of five days followed by a slow tapering of the dosage. Three patients reported immediate relief after the first dose, while ten reported a cessation of cluster attacks after 3.8 (+/− 2.2) days (10).

Since the evidence regarding efficacy and safety of prednisone in the initial treatment of ECH is so limited, ECH patients often suffer from the delayed initiation of a treatment intervention. This trial will provide phase III evidence regarding efficacy and safety of oral prednisone added to first-line agent verapamil in the beginning of a cluster headache episode as compared to monotherapy with verapamil. Furthermore, this trial will also provide evidence regarding long-term outcomes in ECH-patients after prednisone administration.

### Alternative treatment options

Lithium is the only agent approved for the prophylactic treatment of ECH (dosage between 600 mg and 1500 mg/d), despite the lack of a controlled clinical trial [16]. Lithium requires regular plasma concentration and laboratory monitoring due to its various side effects [17]. Therefore it is mentioned in both German and international treatment guidelines only as the 2nd choice when therapy with verapamil fails or if there are contraindications for the use of verapamil. Verapamil is the only substance that was tested in a controlled clinical trial following current standards for evidence-based medicine [5].

Other second-line agents with unproven efficacy in ECH are: pizotifen, methysergide, valproic acid, topiramate, melatonin and gabapentin [18-26]. These substances are used in an off-label-indication. There are no data regarding prophylactic therapy of ECH using triptans on a daily basis: while sumatriptan (100 mg/d) showed no effect on the long term outcome, naratriptan (2,5 – 5 mg/d) and eletriptan (40 mg/d) led to a significant reduction of attack frequency [27-29].

Another treatment option is the local infiltration of the greater occipital nerve with local anesthetics and steroids [30-32]. Two randomized-controlled trials reported a significant reduction in the number of cluster attacks after suboccipital injection of corticosteroid preparations targeting the greater occipital nerve [33,34]. There is no evidence yet as to which corticosteroid preparation, what form of application, or which dosage is the most efficient in the treatment of ECH.

## Methods and design

### Design

PredCH is a prospective, randomized, double-blind, placebo-controlled trial with two parallel interventional arms: all eligible patients with ECH will receive verapamil and will then be 1:1 randomized to the treatment intervention with prednisone or placebo. This multi-center study is being conducted at eight German sites specializing in headache treatment, and aims to recruit a total of 144 patients (see section on sample size calculation below).

### Primary objective

The primary objective of this trial is to assess whether prednisone can reduce the total number of cluster attacks within the first week of treatment compared to a placebo, as documented by the patients in their headache diary. This endpoint follows the “Guidelines for controlled trials of drugs in cluster headache” of the International Headache Society and has been used in previous trials on ECH treatment.

### Secondary objectives

PredCH will also assess the effect of ± prednisone for the following secondary endpoints:

1. Number of cluster attacks from day 1 to day 28 of study participation.

2. Number of cluster attacks from day 7 to day 28 of study participation.

3. Episode abortion (yes/no), defined as no further attacks in the last 3 days before follow-up on day 7 or day 28.

4. Acute medication intake up till day 7; up till day 28.

5. Responder rate (defined as a reduction in the number of daily attacks >50% measured after 7 and 28 days as compared to mean number of attack during the last three days before inclusion).

6. Presence or absence of trigemino-autonomic symptoms (yes/no) after 7 and 28 days:

a) Lacrimation

b) Nasal congestion, rhino rhea

c) Conjunctival injection

d) Ptosis

e) Miosis

f) Facial sweating on side of pain

7. Impact on quality of life, assessed on day −1 and day 28:

a. SF-12 [34]

b. HIT-6 [35]

c. ADS [36]

8. Pain intensity (mean) of cluster attacks in the first seven days and the first 28 days after initial treatment as measured by the Numerical Rating Scale.

9. Tolerability and safety

a. Adverse events, pathological findings on neurological and medical examination, urinary and blood tests, ECG changes.

b. Drug accountability check on day 7 and day 28

c. CGI [37] on day 7 and day 28, compared to baseline.

### Interventions

Patients in the active add-on treatment arm (treatment intervention) are administered oral prednisone as an add-on therapy to the prophylactic treatment with verapamil. Administration accords to Table 1. The dosing regimen is adapted from international treatment guidelines [2-4].

**Table 1:** Dosing scheme of prednisone/placebo

Day 1 – 5	100 mg (5× 20 mg) prednisone/placebo P.O. once daily
Day 6 – 8	80 mg (4× 20 mg) prednisone/placebo P.O. once daily
Day 9 – 11	60 mg (3× 20 mg) prednisone/placebo P.O. once daily
Day 12 –14	40 mg (2× 20 mg) prednisone/placebo P.O. once daily
Day 15 – 17	20 mg (1× 20 mg) prednisone/placebo P.O. once daily

Patients in the placebo arm will receive placebo capsules P.O. (Table 1) as an add-on therapy to the prophylactic treatment with verapamil. Both groups will also receive potassium substitution and pantoprazole 40 mg daily.

Patients in both groups are allowed to use their regular analgesic medication for acute attacks (see also “additional pain medication”) and will receive the basic and additional therapy described below.

Compliance is controlled and documented on day 7 and day 28. In case of a medical emergency the arms can be unblinded.

Prednisone and placebo capsules are produced for this clinical trial at the pharmacy of the University of Heidelberg.

### Basic therapy

In addition to prednisone or a placebo, each patient will be prescribed the standard prophylactic therapy with verapamil P.O., starting on the first day of study participation and following the dosing scheme in Table 2.

**Table 2:** Dosing scheme of verapamil

Day 1 – 3	40 mg - 40 mg - 40 mg
Day 4 – 6	40 mg - 40 mg - 80 mg
Day 7 – 9	40 mg - 80 mg - 80 mg
Day 10 – 12	80 mg - 80 mg - 80 mg
Day 13 – 15	80 mg - 80 mg - 120 mg
Day 16 – 18	80 mg - 120 mg - 120 mg
Day 19 – end of trial	120 mg - 120 mg - 120 mg

If a patient experiences intolerance or adverse effects due to verapamil before reaching the target dosage of 360 mg daily, lower tolerable doses can be continued.

### Additional therapy

Oral potassium (as an effervescent tablet) 1,56 g/d and pantoprazole 40 mg/d P.O. will be prescribed to prevent prednisone side effects such as gastric ulcer and hypokalemia.

### Additional pain medication

The following drugs and maximal daily doses will be allowed:

NSAID: Ibuprofen 800 mg/d; aspirin 500 mg/d; diclofenac 50 mg/d; naproxen 250 mg/d paracetamol 1000 mg/d; metamizole 2000 mg/d. Mixed analgesics: i.e.Thomapyrin (acetylsalicylic acid 250 mg, paracetamol 200 mg and caffeine 50 mg) 1 tablet per day.

Triptans: almotriptan 2× 12,5 mg/d; eletriptan 2× 40 mg/d; rizatriptan 2× 10 mg/d; frovatriptan 2× 2,5 mg/d; naratriptan 2× 2,5 mg/d; sumatriptan 2× 100 mg/d P.O.; 2× 20 mg/d nasal; 2× 6 mg/d SQ; zolmitriptan 2 × 5 mg P.O.; 2× 5 mg nasal.

Ergotamine 2× 2 mg/d P.O.

Lidocaine (4%) nasal 0,5 ml/d.

High flow (6–12 l/min) inhalation of 100% oxygen.

### Estimated timeline

Recruitment will begin in April 2013 and end in March 2015.

### General structure of the study

**Figure 1:**
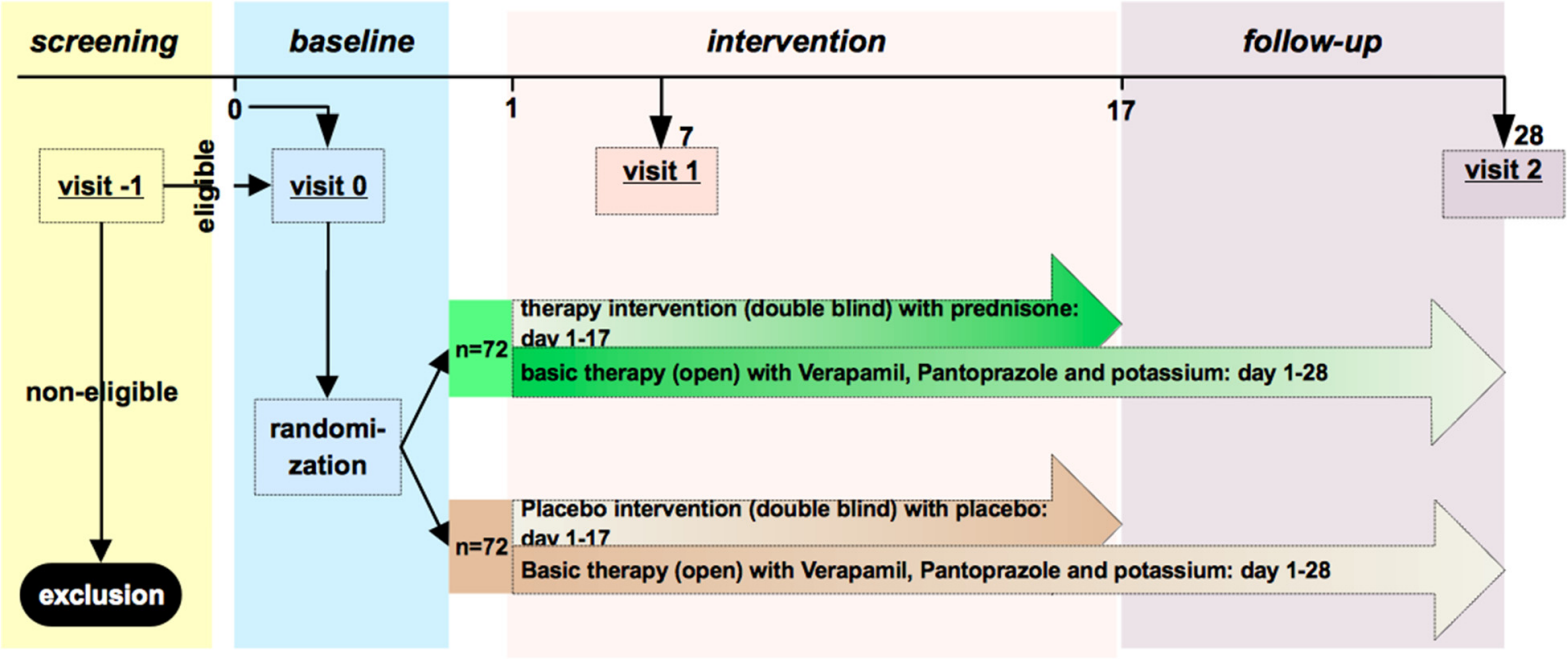
Visit scheme of PredCH (will be provided as separate file).

The duration of the study for each patient is 28 days (including 17 days of treatment/placebo intervention). It consists of 4 visits (see Figure 1) including a screening visit, a randomization visit, and two follow-up visits during which therapy response, safety, and compliance will be assessed.

### Screening visit (“visit −1”)

The screening visit consists of a standardized interview, assessment of eligibility and informed consent. Impairment by ECH is measured with three instruments, evaluating quality of life (SF-12), depression (ADS) and severity of headache (HIT-6). Medical and neurological exams are performed and an ECG and vital parameters are obtained prior to verapamil administration. Urine and blood samples are obtained in order to screen for diabetes, electrolyte disturbances and ongoing systemic infection (e.g. acute urinary tract infection) prior to prednisone therapy. Female patients will also be screened for pregnancy.

### Visit 0 (baseline)

The study patient will be randomized and study medication will be handed out. The headache diary will be explained to the patient. Screening and randomization can take place on the same day.

### Visit 1

Visit 1 will take place seven days after the baseline visit. A standardized interview will be performed as well as a medical and neurological examination. ECG and vital parameters will be documented. Therapy compliance and the headache diary will be controlled and headache frequency evaluated. Adverse events and dropouts from the study will be documented.

### Visit 2

Visit 2 will be performed 28 days after inclusion. In addition to the tests performed in visit 1, impairment of quality of life will be reassessed (HIT-6, ADS and SF-12) and blood tests will be performed in order to evaluate safety.

### Randomization/stratification

Randomization will be conducted using a web-based tool “TenALEA”. Patients will be stratified at randomization according to age (<30 years vs. ≥30 years), sex and participating site.

### Eligibility

#### Inclusion criteria

Male and female patients between 18 and 65 years of age, of legal competence, with sufficient knowledge of written and spoken German, capable of attending regular follow-up visits.

Episodic cluster headache according to the IHS-criteria; patient has had at least one previous episode (bout) before; average duration of previous cluster episodes should have been at least one month; expected duration of cluster period after randomization should be at least one month after initiation of therapy; previous cluster episodes should have ended at least one month prior to inclusion; beginning of ongoing bout of cluster headache should be less than 30 days ago.

#### Exclusion criteria

History of severe allergic diathesis; intolerance or contraindications against verapamil, prednisone, pantoprazole or potassium (effervescent tablet); diabetes mellitus (history and screening); cardiac arrhythmia (second and third degree AV block, SA block, sick sinus syndrome, atrial fibrillation, atrial flutter, WPW-Syndrome, bradycardia with HR <50/min); arterial hypotension (systolic pressure <90 mmHg); hypertension (>180 mmHg); history of GIT-ulceration; severe osteoporosis; glaucoma; tuberculosis; current viral (e.g. HSV, VZV, herpetic keratitis) fungal, parasitic or bacterial infections (laboratory screening); poliomyelitis; lymphadenitis following BCG vaccination; acute urinary tract infection; chronic cluster headache; prednisone or verapamil medication less than 30 days before inclusion into the study.

Participation in a different clinical trial less than 30 days before inclusion; previous inclusion into PredCH; parallel participation in a different clinical trial; ongoing substance/alcohol abuse/dependence; history of psychiatric disease with risk of suicide; history of severe chronic or terminal illness; HIV.

Chronic disease which causes impairment of absorption, metabolism, secretion of study medication (e.g. chronic inflammatory bowel disease, renal insufficiency); chronic hepatic disease (lab screening: 2–3 fold increase above normal in liver function test); history of neuromuscular disease (e.g. myasthenia gravis; Duchenne muscular dystrophy, Lambert-Eaton-Syndrome); nursing; pregnancy (pregnancy testing); fertile women with insufficient contraception; absence of consent.

### Excluded therapies and medications

Concomitant medication/nonmedical treatment will be documented.

The following substances cannot be taken during trial, due to possible interactions with study medication: beta antagonists; cardiac glycosides; antiarrhythmic agents (flecainide, disopyramide, quinidine); oral anticoagulants (dabigatran, rivaroxaban, abixaban, vitamin-K antagonists); non-depolarizing neuromuscular blockers; atropine and other anticholinergic agents; praziquantel; chloroquine; hydroxychloroquine; mefloquine; somatotropin; protirelin; inhalational anesthetics; SSRIs and SNRIs; MAO-Is; lithium; laxatives; doxyorubicine; oral antihyperglycemic agents; antacids; indometacin; postmenopausal hormone substitution; colchicine; ethanol; other inhibitors of cytochrome P450 isoenzyme 3A4 such as anti-fungal azoles (e.g. clotrimazole, ketoconazole or itraconazole); protease inhibitors (e.g. ritonavir or indinavir); macrolides; cimetidine; antidepressants; inducers of cytochrome P450 isoenzyme 3A4 such as phenytoin; rifampicin; phenobarbital; carbamazepine; urikosuric agents (sulfinpyrazone); Hypericum perforatum (St John’s wort); benzodiazepines and other anxiolytic agents (e.g. buspirone); substrates of cytochrome P450 isoenzyme 3A4 such as antiarrhythmic agents (e.g. amiodarone); statins (e.g. simvastatin > 20 mg/d or atorvastatin > 40 mg/d); midazolam; ciclosporin, everolimus; sirolimus; tacrolimus; theophylline; prazosin; terazosin.

The following substances which can be used as prophylactic treatment of ECH cannot be taken within 30 days before inclusion into study: methysergide; lithium; topiramate; pizotifen; valproic acid; melatonin; gabapentin.

### Assessment of representativeness

Patients screened for PredCH, who are not included in the study will be registered and documented (date, age, sex, reason for exclusion) in order to assess representativeness of the included patient group.

Patients can always withdraw their consent to participate in the study without stating a reason.

### Statistical analysis and sample size considerations

#### Study populations

All randomized patients who have taken the study medication at least once will be included in the safety assessment group that accords to the treatment group to which they were assigned. Similarly, the intention-to-treat-analysis (ITT) population will be based on patients who have taken the study medication once, but analyses will be done according to randomization allocation. The per-protocol (PP) population excludes patients with protocol violations as defined by the sponsor or the responsible biometrician prior to unblinding.

#### Efficacy

The primary endpoint in the efficacy assessment will be the total number of cluster attacks within the first week of treatment.

The null hypothesis that both intervention groups have the same mean number of attacks will be tested against the (two-sided) alternative hypothesis that there will be a difference in the mean number of attacks between treatment intervention group and placebo group after one week. The confirmatory analysis will be performed in the ITT population.

A generalized linear model will be used to adjust for the stratification variables of sex, age (< 30 years vs. ≥ 30 years) and participating site. The null hypothesis will be rejected when the two-sided p-value of the Wald-test for the intervention group variable is equal or less than the significance level α = 0.05. The average mean difference in the primary endpoint is assumed to be clinically relevant when the mean number of cluster attacks is more than 30% lower in the add-on prednisone group than in the add-on placebo group.

Additionally there will be explorative sensitivity analyses – e.g. in the PP populations. Similarly, secondary endpoints will be analyzed exploratively using appropriate methods of descriptive and inferential statistics. Details will be described in a statistical analysis plan.

#### Safety

Analysis of tolerability and safety will be performed for all patients who took at least one dose of study medication (safety group). During screening and follow up visits patients will participate in a safety assessment which will examine for pathological findings in medical and neurological examination and laboratory tests, abnormal vital parameters, adverse events and suspected adverse effects from study medication. The safety report will analyze differences over time as well as between both intervention groups. Severe adverse events will be reported as single case histories.

#### Sample size and power

The sample size calculation is based on the parametric evaluation of a two group comparison using a *t*-test though a more complex statistical model will be used as primary test. Based on data from the literature [5] we estimate the average frequency of headache attacks as 8.25 (with a standard deviation (SD) of 4.2) for 5 days and assume equal SDs in both treatment groups. Requiring α = 0.05 (two-sided) while aiming at a comparison-wise power of 1-β = 0.9 (a higher power was chosen to address the problem that a more complex statistical analysis will be used), a sample size of n = 2 × 61 = 122 patients is necessary for the ITT analysis to detect a mean difference of 2.5 in average frequencies of headache attacks during the first 5 days between treatment by oral prednisone vs. placeobo. In order to address a potential drop-out rate of 15% overall, another 22 patients have to be randomized. Thus, the total maximum sample size is n = 2 × 72 = 144 patients.

#### Quality assurance/monitoring

The Center for Clinical Trials, Essen (“Zentrum für Klinische Studien Essen” (ZKSE); http://www.zkse.de) will be responsible for the data quality management and monitoring of this trial. As a full member of the national Network of Coordinating Centres for Clinical Trials (KKS Network; http://www.kks-netzwerk.de/), the ZKSE has adapted all harmonized standard operation procedures to the local conditions.

According to the guidelines of GCP, the sponsor is required to select investigators and trial sites as well as review their qualification and the availability of appropriate resources. These will be assessed in a pre-trial visit. The monitor’s report will be distributed to the sponsor, clinical project management and the funding agency. Finally, the sponsor will decide if a site and the related investigators are suitable.

### Informed consent, ethics, data safety monitoring board

#### Informed consent

Prior to inclusion into the study, written informed consent must be obtained from each patient and participation in the trial and written consent will be documented in the patient’s file. The patient must be informed generally about the study, alternative treatment options, risks and benefits from the study, as well as his/her right to withdraw consent.

Each patient who has been entered in the study will receive further prophylactic treatment with verapamil, lithium or topiramate and will be offered a follow-up visit 2 months after the study, even should he/she drop out.

#### Ethics/legal approval

Documented approval has been obtained from the responsible ethics committees at all participating study centers. A list of the responsible ethics committees is provided below. The study conforms to the Declaration of Helsinki, the German legal regulations of the Medicinal Products Act (“Arzneimittelgesetz”) and the current version of the ICH-GCP guidelines.

### List of the responsible ethics commitees

• Ethik-Kommission der Medizinischen Fakultät der Universität Duisburg-Essen

• Ethik-Kommission des Landes Berlin

• Ethik-Kommission der Medizinischen Fakultät der Martin-Luther-Universität Halle-Wittenberg

• Ethik-Kommission an der Medizinischen Fakultät der Friedrich-Schiller-Universität Jena

• Ethik-Kommission bei der LMU München

• Ethik-Kommission der Ärztekammer Westfalen-Lippe und der Medizinischen Fakultät der WWU Münster

• Ethik-Kommission der Ärztekammer Nordrhein

• Ethik-Kommission der Ärztekammer Schleswig-Holstein

### Data safety monitoring board

There will be annual controls by the data safety monitoring board, which consists of a neurologist, a cardiologist and a biometrician. This committee will analyze all severe adverse events, infections, drop-outs due to adverse medical effects and mortalities.

## Discussion

PredCH will assess efficacy and safety of prednisone in prophylactic treatment of episodic cluster headache in addition to first line therapy with verapamil in a double-blind controlled trial. Eligible patients will be randomized 1:1 to add-on oral prednisone or add-on placebo with dosages corresponding to international recommendations.

This multi-center trial aims to provide the evidence on efficacy and safety for the initial treatment of ECH, so that patients will be able to receive therapy much faster, both in an in- and outpatient setting. Furthermore, the trial will provide insight into the long-term outcome of ECH after prednisone administration.

## Abbreviations

ADS: Allgemeine Depressionsskala: General Depression Scale – a psychometric self-assessment screening test for depression; CGI: Clinical Global Impression- a scale that helps evaluating effects of therapeutic intervention, regarding severity of disease, improvement efficacy and adverse effects of therapy; ECH: Episodic cluster headache; ECG: Electrocardiogram; GCP: Good Clinical Practice; GIT: Gastrointestinal tract; HIT-6: Headache Impact Test – a six-item questionnaire for screening and monitoring outcomes of patients with headache; HR: Heart rate; HSV: Herpes simplex virus; ICD-10: International Classification of Diseases 10th revision; ICH: International Conference on Harmonisation; IHS: International Headache Society; IMIBE: Institut für Medizinische Informatik, Biometrie und Epidemiologie: Institute for Medical Informatics, Biometrics and Epidemiology; ITT: Intent-to-treat; I.V.: Intravenous administration; MAO-I: Inhibitors of monoamine oxidase; NSAID: Non-steroidal anti-inflammatory drug; PI: Principal investigator; P.O.: Per os, oral administration; SQ: Subcutaneous injection; SF-12: 12-item short form questionnaire: a questionnaire to assess quality of life; SSRI: Selective serotonin reuptake inhibitor; SNRI: Serotonin norephedrine reuptake inhibitor; VZV: Varizella zoster virus; WPW: Wolff-Parkinson-White-syndrome; ZKSE: Zentrum für Klinische Studien Essen: Center for Clinical Trials Essen.

## Competing interests

The authors declare that they have no competing interests.

## Authors’ contributions

DH designed the study protocol, acquired the necessary funding, and participates in the coordination and analysis of the study. MO designed the study protocol, acquired the necessary funding and participates in the coordination and analysis of the study. CO participated in the design of the study and the statistical analysis. AS designed the study protocol, acquired the necessary funding and is responsible for methodological and statistical considerations related to the trial. JB drafted the manuscript and participates in the coordination and analysis of the study. HCD participated in the design and conceptualization of the study. All authors read and approved the final manuscript.

## Authors’ information

DH is from the Department of Neurology at the University Hospital Essen. She is a fellow in neurology.

MO is from the Department of Neurology at the University Hospital Essen. He is a consultant in neurology.

CO works at both the Center for Clinical Trials (ZKSE) and the Institute for Medical Informatics, Biometry and Epidemiology of the University Hospital Essen.

AS is head of the Unit Biometry and Bioinformatics at the Institute for Medical Informatics, Biometry and Epidemiology of the University Hospital Essen and is head of Biometry at the Center for Clinical Trials (ZKSE).

HCD is the Chair of the Department of Neurology and the Headache Center at the University Hospital Essen and Professor of Neurology at the University Duisburg-Essen.

JB is from the Department of Neurology at the University Hospital Essen. He is a 2^nd^ year resident.

## List of participating centers and local PIs

Universitätsklinikum Essen, Klinik und Poliklinik für Neurologie, Hufelandstr. 55, 45122 Essen, Germany, Principal Investigator: PD Dr. M. Obermann.

Ludwig-Maximilians-Universität München, Klinikum Großhadern, Neurologische Klinik, Marchioninistr. 15, 81377 München, Germany, Principal Investigator: Prof. Dr. A. Straube.

Universitätsklinikum Jena, Klinik für Neurologie, Erlanger Allee 101, 07747 Jena, Germany, Principal Investigator: Prof. Dr. O.W. Witte.

Universitätsklinikum Münster, Klinik für Neurologie, Albert-Schweitzer-Campus 1, Gebäude: D5, Domagkstraße 5, 48149 Münster, Germany, Principal Investigator: PD Dr. M. Marziniak.

Neurologisch-verhaltensmedizinische Schmerzklinik Kiel, Heikendorfer Weg 9–27, 24149 Kiel, Germany, Principal Investigator: Prof. Dr. H. Göbel.

Kliniken der Stadt Köln, Neurologische Klinik, Ostmerheimer Str. 200, 51109 Köln, Germany, Principal Investigator: Dr. Lutz Pageler.

Martin-Luther-Universität Halle-Wittenberg, Klinik und Poliklinik für Neurologie, Ernst-Grube-Str. 40, 06097 Halle, Germany, Principal Investigator: Dr. Torsten Kraya.

Charité, Klinik für Neurologie, Centrumsleitung, CC 15&16, Charitéplatz 1, 10117 Berlin, Germany, Principal Investigator: PD Dr. Uwe Reuter.

## Pre-publication history

The pre-publication history for this paper can be accessed here:

http://www.biomedcentral.com/1471-2377/13/99/prepub
